# Plot size matters: Toward comparable species richness estimates across plot‐based inventories

**DOI:** 10.1002/ece3.8965

**Published:** 2022-06-12

**Authors:** Jeanne Portier, Florian Zellweger, Jürgen Zell, Iciar Alberdi Asensio, Michal Bosela, Johannes Breidenbach, Vladimír Šebeň, Rafael O. Wüest, Brigitte Rohner

**Affiliations:** ^1^ Swiss Federal Institute for Forest, Snow and Landscape Research WSL Birmensdorf Switzerland; ^2^ Centro Superior de Investigaciones científicas Instituto Nacional de Investigación y Tecnología Agraria y Alimentaria Centro de Investigación Forestal Madrid Spain; ^3^ Faculty of Forestry Technical University in Zvolen Zvolen Slovakia; ^4^ Forest Research Institute Zvolen National Forest Centre Zvolen Slovakia; ^5^ 56624 Division of Forestry and Forest Resources Norwegian Institute of Bioeconomy Research Ås Norway

**Keywords:** biodiversity, monitoring program, National Forest Inventory, rarefaction curve, species richness, species–area relationship

## Abstract

To understand the state and trends in biodiversity beyond the scope of monitoring programs, biodiversity indicators must be comparable across inventories. Species richness (SR) is one of the most widely used biodiversity indicators. However, as SR increases with the size of the area sampled, inventories using different plot sizes are hardly comparable. This study aims at producing a methodological framework that enables SR comparisons across plot‐based inventories with differing plot sizes. We used National Forest Inventory (NFI) data from Norway, Slovakia, Spain, and Switzerland to build sample‐based rarefaction curves by randomly incrementally aggregating plots, representing the relationship between SR and sampled area. As aggregated plots can be far apart and subject to different environmental conditions, we estimated the amount of environmental heterogeneity (EH) introduced in the aggregation process. By correcting for this EH, we produced adjusted rarefaction curves mimicking the sampling of environmentally homogeneous forest stands, thus reducing the effect of plot size and enabling reliable SR comparisons between inventories. Models were built using the Conway–Maxell–Poisson distribution to account for the underdispersed SR data. Our method successfully corrected for the EH introduced during the aggregation process in all countries, with better performances in Norway and Switzerland. We further found that SR comparisons across countries based on the country‐specific NFI plot sizes are misleading, and that our approach offers an opportunity to harmonize pan‐European SR monitoring. Our method provides reliable and comparable SR estimates for inventories that use different plot sizes. Our approach can be applied to any plot‐based inventory and count data other than SR, thus allowing a more comprehensive assessment of biodiversity across various scales and ecosystems.

## Graphical abstract

Species richness is a commonly used biodiversity indicator. Because it strongly depends on the area sampled, comparability of species richness estimates across inventories using different plot sizes is not directly possible. We provide a methodological framework based on rarefaction curves to tackle this challenge.

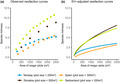

## 1 INTRODUCTION

As human activities continue to trigger rapid climate change as well as high biodiversity turnover and extinction rates, monitoring biodiversity has become a high priority for the scientific community (IPBES, 2019). The many monitoring programs that were developed over the last decades at local‐to‐national scales provide valuable information on the state and trends of biodiversity. Merging these existing datasets and comparing outputs beyond the local or national scale are essential to study biodiversity in a more comprehensive way in order to reliably inform decision and policy makers (Lengyel et al., 2008; Winter et al., 2008). In fact, producing robust outputs from merged datasets is a key objective of main European policy strategies (European Commission, 2021). However, it is often hampered by the fact that sampling designs, field protocols, and estimation procedures can differ strongly from one inventory to another. Thus, comparability of the many existing datasets constitutes a major challenge. For example, sampling designs do not always follow a spatially systematic (gridded) design, can have different inclusion probabilities, or different plot sizes. Tackling these differences is challenging but important because sampling designs can strongly affect derived biodiversity indices. Disregarding sampling design effects can thus lead to deceptive results. With an increasing demand for comparable information on biodiversity, the need for developing methods allowing inference across inventories becomes urgent.

Species richness (SR), here referring to the number of species occurring in a given area, is an intuitive diversity index commonly used as a proxy for biodiversity components. It is a highly important ecological indicator shown to be a key driver of ecosystems’ resilience (Oliver et al., 2015). For instance, forests with higher SR suffer less from the impact of disturbances and are thus better able to retain their carbon stocks, which is a crucial mitigation strategy in the face of climate change (Guyot et al., 2016; Silva Pedro et al., 2015). It is, however, surprisingly difficult to provide robust estimates of SR from plot‐based monitoring data (Gotelli & Colwell, 2011). The size of monitored plots directly affects how many species can be found (Hill et al., 1994) because SR increases non‐linearly with the area of the sampling units (Dengler, 2009; Gotelli & Colwell, 2001). For this reason, SR estimates should always be provided along with the corresponding plot size. Problems arise when, for instance, one wishes to compare the tree SR of two different countries, but one country uses 250 m² forest plots as sampling units while the other relies on 500 m² plots. A lower reported mean SR across all plots in the first country may be an ecological fact, or it may instead be rooted in the smaller plot size. Avoiding such uncertainties is utterly important because political decisions regarding biodiversity are based on indices such as the mean SR, although they are often not directly comparable across inventories. A simple solution to the problem exists when the location of each sampled individual is known within the plots. In this case, the size of larger plots can artificially be decreased to match the size of the smallest plots. Following our previous example, the size of the second country’s plots could be artificially decreased from 500 to 250 m², allowing for a direct comparison of its SR to that of the first country. However, not only is the location of every individual rarely available in monitoring programs, but this process leads to an immense information loss: larger plots would always need to be downscaled to the smallest sampled plot size. In this context, obtaining reliable comparisons of SR between inventories using different plot sizes must involve upscaling SR to larger areas. We suggest estimating species–area relationships from rarefaction curves built on aggregated plots as a robust approach to do so, allowing in our previous example to compare the tree SR of both countries for the same given area.

Species–area relationships are a well‐established concept in ecology and biodiversity science (e.g., Dengler, 2009; Gotelli & Colwell, 2001; Stein et al., 2014; Tittensor et al., 2007). They are commonly built from a nested design, where the area of a plot is gradually increased in a continuous manner and SR is calculated accordingly (Dengler et al., 2020; Gotelli & Colwell, 2001). In inventories relying on a network of independent sample plots, increasing area is achieved by aggregating non‐contiguous plots. The relationship between the area of aggregated plots and their corresponding SR is referred to as sample‐based rarefaction curves (Crist & Veech, 2006; Gotelli & Colwell, 2001; Steinmann et al., 2011). Plot size determines how many plots must be aggregated to reach a given area. In our previous example, the mean SR of the second country for an area of 500 m² simply corresponds to the mean SR across all individual sampled plots. However, it corresponds in the first country to the mean SR of many combinations of two randomly aggregated plots. It is crucial to note that any two plots of the first country that together make up the 500 m^2^ can potentially be located in very different habitats and subject to different environmental conditions. This makes it likely to find different sets of species, generally leading to a higher combined SR in two different plots of smaller size than in one homogeneous plot of larger size. In other words, the smaller single plots are, the more different environments are likely to be sampled for a given aggregated area, and the faster SR accumulates with area in rarefaction curves (Gotelli & Colwell, 2001; Steinmann et al., 2011). As a consequence, this prevents direct comparisons of rarefaction curves across inventories that use different plot sizes. Here, we hypothesize that we can produce comparable, adjusted rarefaction curves by controling for what drives the difference in species found in aggregated plots: environmental heterogeneity and spatial configuration.

Environmental heterogeneity (EH) is a key determinant of the shape of sample‐based rarefaction curves. Simply because it is likely that a wide range of environmental conditions is encountered when aggregating independent plots, the probability for additional species to occur rises (Drakare et al., 2006; Stein et al., 2014; Steinmann et al., 2011). Plots located in regions that differ in terms of climate, topography, soil conditions, or vegetation structure are likely to also differ in terms of species. While there is a growing consensus that EH across aggregated plots increases SR (Stein et al., 2014), disentangling this effect from the effect of plot size remains challenging (Steinmann et al., 2011). The second important aspect that affects the shape of rarefaction curves is the spatial configuration of aggregated plots, i.e., the geographic distance between plots that have been aggregated and the resulting spatial extent of the total area that has been sampled (Güler et al., 2016). This can represent legacy effects such as demographic processes, dispersal limitation, colonization probabilities or speciation, and extinction processes that all affect the capacity of a given species to occur at a given location (Drakare et al., 2006). In order to compare SR across inventories that use different plot sizes, the key challenge is therefore to develop a method accounting for EH and the spatial configuration of aggregated plots when building rarefaction curves.

**FIGURE 1:**
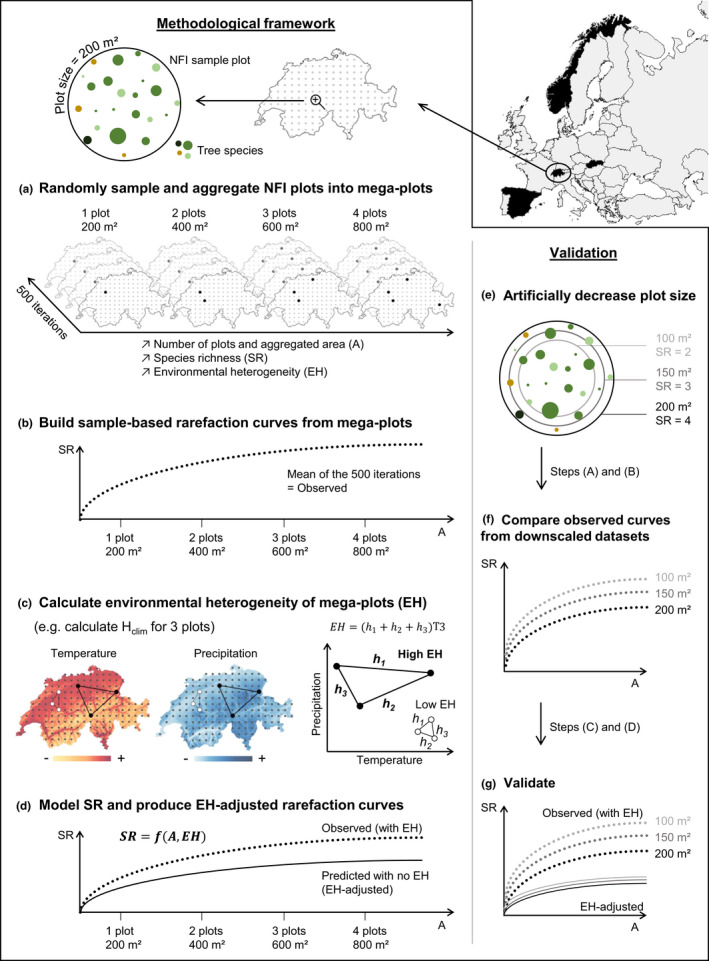
Conceptual figure describing the methodological steps taken to remove the effect of EH introduced in the aggregation of plots. These steps were applied independently to each country, but are shown in this figure for Switzerland as an example. Each step is further described in the Materials and Methods section

In this study, we propose a methodological framework for modeling SR as a function of area, EH, and spatial configuration, thus accounting for a variety of ecological factors known to affect SR (Figure 1). Our approach artificially removes the plot size‐dependent effects of EH and spatial configuration that are introduced when aggregating plots. The resulting adjusted rarefaction curves are then independent of plot size and represent the relationship between SR and area as if the aggregated plots were subject to similar environmental conditions. In other words, the proposed methodological framework mimics a situation where species were recorded from a single, large, environmentally homogeneous plot. Although investigating the effect of EH on rarefaction curves would be another interesting avenue itself, here we focus on developing a method to obtain reliable comparisons of SR estimates between inventories with different plot sizes. The wealth of data collected within National Forest Inventories (NFIs) and the growing relevance of the NFI community for large‐scale biodiversity reporting provide a valuable opportunity to develop and test this approach (Corona et al., 2011; Vidal, Alberdi, Redmond, et al., 2016). NFIs are conducted in most European countries (Tomppo et al., 2010; Vidal, Alberdi, Redmond, et al., 2016), but each differs with respect to sampling design settings such as plot size, field protocols, or estimation procedures (Chirici et al., 2011; Winter et al., 2008). We gathered NFI tree species occurrence data from Norway, Slovakia, Spain, and Switzerland, covering a wide ecological and bioclimatic gradient extending over seven of the ten European biogeographical regions (European Environment Agency, 2016). NFI data are also a rare case where the location of individuals within plots is known, bringing a unique opportunity to validate our approach, which is essential to demonstrate its general applicability for other monitoring programs and their data.

## 2 MATERIALS AND METHODS

### 2.1 National Forest Inventory data

Most NFIs consist of country‐specific networks of independent, systematically distributed sample plots and are representative of the forested area of each country (Tomppo et al., 2010; Vidal, Alberdi, Hernández Mateo, 2016). As our approach, based on NFI data from Norway, Slovakia, Spain, and Switzerland, relies on sample plot size, we excluded all NFI plots for which size was reduced by a road, a river, or any other natural or anthropogenic barrier. In Spain, only plots located in the Peninsula were used. The total number of selected plots and species in each country is presented in Table 1. We defined SR at the plot level as the number of tree species recorded in a plot.

**TABLE 1:** Characteristics of the NFI data used in the analyses for Norway, Slovakia, Spain, and Switzerland. These characteristics do not correspond to the full original NFI dataset of each country, but to the NFI plots used in our analyses after we performed data harmonization steps

Country	Plot size (m²)	Number of plots	Stratification	Total number of species	Inventory cycle
Norway	250	9844	Yes	29	2014–2018
Slovakia	500	1277	No	56	2015–2016 (NFI2)
Spain	300	65,236	Yes	126	1997–2006 (NFI3)
Switzerland	200	5361	No	56	2009–2013 (NFI4)

We performed further data harmonization steps to control for the main sampling design differences between countries. Some NFI plot configurations are based on concentric circles associated with different diameter at breast height (DBH) thresholds (e.g., Spain, Slovakia, and Switzerland), on fixed‐area plots (e.g., Norway) or on angle count sampling. To minimize the effect of DBH thresholding and to base our analyses on fixed areas, we selected only one circle in the NFIs that use concentric circles. In Switzerland and Slovakia, we retained the 200 and 500 m² circles, respectively, in both cases associated with a DBH threshold of 12 cm. In Spain, we used the 10‐m‐radius circle (314 m², DBH threshold = 12.5 cm) that we cropped at 300 m² to facilitate subsequent analyses requiring 50 m² increments. In Norway (plot size = 250 m²), we removed trees with a DBH <12 cm to harmonize the DBH threshold throughout the countries. Note that NFI data used in this study are not necessarily the ones used for international reporting, such as in the State of Europe’s Forests report (FOREST EUROPE, 2020). All analyses were performed in R.3.6.3 (R Core Team, 2020).

### 2.2 Building sample‐based rarefaction curves (Figure 1a,b)

We used these NFI data to build sample‐based rarefaction curves (Gotelli & Colwell, 2001, 2011), representing the relationship between SR of aggregated plots and area. To this end, we randomly sampled and aggregated independent NFI plots within each country (Figure 1a) to incrementally increase area from that of a single plot to a maximum of 10,000 m² (1 ha). We did not limit the spatial extent of the aggregated plots to smaller regions within countries as the goal was to compare the SR of whole countries, as per the State of Europe’s Forest report (FOREST EUROPE, 2020). As plot size differed between countries, the number of plots required to reach 1 ha varied between countries (from 20 in Slovakia to 50 in Switzerland). Aggregated plots are hereafter referred to as “mega‐plots.”

Contrary to Slovakia and Switzerland where NFI plots are evenly distributed on an equidistant grid, the grid resolution of Norway (Breidenbach et al., 2020) and Spain (Alberdi et al., 2017) differs between regions (i.e., sampling strata). In these countries, the random sampling for generating mega‐plots was adjusted to account for region‐specific sampling intensity, such that the chances of a plot to be drawn in the aggregation process were reciprocally proportional to the grid density. That means, plots in a stratum with a wider sampling grid had a larger chance to be drawn than plots in a stratum with a denser sampling grid. The random sampling was repeated 500 times, resulting for each country in 500 mega‐plots per aggregation step (Figure 1a). We calculated the SR of mega‐plots at each step. Mega‐plots were used to build country‐specific sample‐based rarefaction curves (Figure 1b), representing the relation between the average SR over the 500 equal‐sized mega‐plots, and the size of these mega‐plots (Crist & Veech, 2006; Gotelli & Colwell, 2001).

### 2.3 Quantifying the EH contained in mega‐plots (Figure 1c)

We used a broad set of environmental variables representing climate, topography, soil, and stand structure to cover the main factors shaping spatial patterns of SR (Field et al., 2009). Climate data were downloaded from Karger et al. (2017). We included mean annual precipitation and mean growing season temperature based on monthly data for the years 1979–2013. Topography variables were derived from a pan‐European digital elevation model (DEM) with a spatial resolution of 25 m (EU‐DEM, 2018). We considered slope inclination and Beers aspect, the latter being an ecologically meaningful index ranging from 0 (southwestern slopes, warm) to 2 (northeastern slopes, cold). Beers aspect was calculated from aspect shifted by a 45 degrees angle (Beers et al., 1966):

(1) $$\text{Beers}\;\text{aspect}=1+\cos\left(45‐\text{aspect}\right).$$

Soil properties were represented by topsoil pH. For Spain and Slovakia, we downloaded topsoil pH data from the European Soil Database, containing maps of topsoil properties based on the Land Use and Cover Area frame Survey (LUCAS) (Ballabio et al., 2019). This database does not provide data for Norway and Switzerland, for which we downloaded topsoil pH from SoilGrids, a global grid of soil information (Hengl et al., 2017). We further used basal area per hectare (BA) as an indicator of forest stand structure, representative of attributes such as stand density, light conditions, and competition at the local scale.

EH measures were derived from these variables for each mega‐plot to depict the differences in environmental conditions representative of climate (*H*_clim_, described by annual precipitation and mean growing season temperature), topography (*H*_topo_, described by slope and Beers aspect), soil (*H*_soil_, described by pH), and BA (*H*_BA_) across the aggregated plots. EH measures were calculated in three steps. First, the scaled value of each environmental variable was extracted for each plot. Second, within each mega‐plot and independently for each EH measure (i.e., *H*_clim_, *H*_topo_, *H*_soil_, and *H*_BA_), we calculated the pairwise Euclidean distances between the values of the environmental variables of each pair of aggregated plots (Figure 1c). The number of pairwise distances for a mega‐plot made of *n* aggregated plots is (*n*∗(*n* − 1))/2. Pairwise distances were calculated as:

(2) $$dist_{i,j}=\sqrt{{\left(Variable1_{i}‐Variable1_{j}\right)}^{2}+{\left(Variable2_{i}‐Variable2_{j}\right)}^{2}}$$

where *dist_i_*_,_ *_j_* represents the Euclidean distance between the scaled values of environmental variables 1 and 2 (e.g., precipitation and temperature for *H*_clim_) of plot *i* and plot *j*. For *H*_soil_ and *H*_BA_ that both only contain one variable (soil pH and BA, respectively), the same formula using only *Variable1* was applied. Third, the final EH measures of each mega‐plot *k* were calculated as the mean of all pairwise Euclidean distances between the *n* plots aggregated:

(3) $${\text{EH}}_{k}=\frac{\sum_{1}^{n}\left(dist_{1,2}+\cdots +dist_{n‐1,n}\right)}{\left(n\cdot \left(n‐1\right)\right)/2}.$$

The larger an EH measure of a given mega‐plot is, the more environmentally different the aggregated plots composing this mega‐plot are. An additional EH measure representing the spatial configuration of aggregated plots was calculated to reflect that species composition does not depend solely on climate, topography, soil, and BA conditions. First, we calculated *H*_geo_ using latitude and longitude as input variables in Equation (2), representing the geographic distance between aggregated plots. This geographic distance, in addition to controling for spatial autocorrelation in species composition, is considered a proxy for legacy effects and other environmental factors that can affect species composition. Consequently, the information contained in *H*_clim_, *H*_topo_, *H*_soil_, and *H*_BA_ is likely to be related to *H*_geo_. To guarantee the independence of predictor variables, we quantified this repeated information by fitting a linear regression performed with the *lm* function:

(4) $$H_{{\text{geo}}_{k}}=\beta_{0}+\beta_{1}\cdot H_{{\text{clim}}_{k}}+\beta_{2}\cdot H_{{\text{topo}}_{k}}+\beta_{3}\cdot H_{{\text{soil}}_{k}}+\beta_{4}\cdot H_{{\text{BA}}_{k}},$$

where *k* stands for mega‐plots. Although in reality, geographic distance is not a result of environmental conditions, this model was built to calculate H_res_, which was then defined as the residuals of the above‐described regression ($H_{{\text{res}}_{k}}=H_{{\text{geo}}_{k}}‐\hat{H_{{\text{geo}}_{k}}}$). H_res_ represents the residual EH captured by H_geo_ once the effect of climate, topography, soil, and BA were accounted for. All EH variables were then standardized to allow comparisons of effect sizes.

### 2.4 Modeling SR as a function of area and EH (Figure 1d)

The SR distribution of each country was underdispered (i.e., less variation in the data than expected under a Poisson distribution ‐ Figure A1). We therefore assumed that SR followed a Conway–Maxwell–Poisson (CMP) distribution, which is a two‐parameter extension of the Poisson distribution that is able to model underdispersed count data (Sellers & Shmueli, 2010; Shmueli et al., 2005). The CMP distribution relies on a parameter $\lambda_{\text{CMP}}$ > 0 and on a dispersion parameter ν ≥ 0. When ν = 1, the CMP distribution is equivalent to a Poisson distribution. Values of ν < 1 correspond to overdispersion, and values of ν > 1 to underdispersion (Shmueli et al., 2005). The SR of mega‐plots *k* was thus specified as:

(5) $${\text{SR}}_{k}∼\text{CMP}\left(\lambda_{k},\nu \right).$$

Independently for each country, we performed a CMP regression on the whole set of mega‐plots based on maximum‐likelihood estimation using the *glm.cmp* function of the “COMPoissonReg” package (Sellers & Raim, 2016). This implementation allows a simultaneous estimation of ${\hat{\lambda }}_{k}$ and $\hat{\nu }$, which later on can be used to estimate the mean predicted SR (see below). Rather than directly on SR_k_, the *glm.cmp* function performs the regression on ${\hat{\lambda }}_{k}$. We assumed ν to be constant, i.e., ν was not made dependent on the variables used in the model.

Explanatory variables included area of mega‐plots, *H*_clim_, *H*_topo_, *H*_soil_, *H*_BA_, and *H*_res_. The power law function has been shown to be well suited to describe the relationship between SR and area (Dengler, 2009; Dengler et al., 2020). Therefore, we formulated the CMP model as:

(6) $$\lambda_{k}=A_{k}^{\beta_{1}}\cdot e^{\left(\beta_{0}+\beta_{2}\cdot H_{{\text{clim}}_{k}}+\beta_{3}\cdot H_{{\text{topo}}_{k}}+\beta_{4}\cdot H_{{\text{soil}}_{k}}+\beta_{5}\cdot H_{{\text{BA}}_{k}}+\beta_{6}\cdot H_{{\text{res}}_{k}}\right)},$$

where *A_k_* represents the area of mega‐plot *k*, and *β*_0_ to *β*_6_ are the parameters to be estimated. ${\hat{\mathrm{SR}}}_{k}$ was then approximated using the following equation (Sellers & Shmueli, 2010; Shmueli et al., 2005):

(7) $${\hat{\mathrm{SR}}}_{k}\approx {\hat{\lambda }}_{k}^{1/\hat{\nu }}‐\frac{\hat{\nu }‐1}{2\hat{\nu }}.$$

We used the country‐specific models to predict SR along the area gradient while setting all EH measures (*H*_clim_, *H*_topo_, *H*_soil_, *H*_BA_, and *H*_res_) in Equation (6) to the value corresponding to no EH and no geographic distance, i.e., to the EH value of mega‐plots made of one plot (Figure 1d). As we standardized the EH measures, the absence of EH corresponds to the standardized equivalent of zero for *H*_clim_, *H*_topo_, *H*_soil_, and *H*_BA,_ and no geographic distance corresponds to the standardized equivalent of $‐\beta_{0}$ following Equation (4) for *H*_res_. For simplicity, this set of values—including that of *H*_res_—will be hereafter referred to as “no EH.” With this step, we aimed at artificially reproducing the aggregation of plots located in an environmentally homogenous forest stand. We refer to these predicted curves as EH‐adjusted rarefaction curves. In each country, observed and EH‐adjusted rarefaction curves were then compared (Figure 1d).

### 2.5 Validation with downscaled datasets (Figure 1e‐g)

As the investigated NFIs record the distance of each sampled tree to the plot center, we could artificially reduce the size of plots by removing outermost trees to fit any desired new radius. Downscaled datasets with reduced radii were created independently in each country using plot sizes ranging from 100m² to the original plot size (Figure 1e). Different datasets sampling the exact same locations but with different plot sizes can show how plot size affects the shape of rarefaction curves. Additionally, they provide an opportunity to validate our method. As the goal is to obtain reliable SR comparisons between inventories using different plot sizes, applying our method to the downscaled and original datasets within a country should deliver the same EH‐adjusted rarefaction curves for the method to be successful. The same previously described methodological steps – generating mega‐plots and modeling SR (Figure 1a‐d) – were therefore applied independently to each downscaled dataset (Figure 1e). For each dataset of each country, 95% bootstrap prediction intervals were calculated on the predicted SR by re‐fitting models over 5000 iterations where datasets were randomly re‐sampled with replacement. In each country, EH‐adjusted rarefaction curves extracted from the downscaled and full datasets were compared (Figure 1f,g). The removal of the effect of EH was considered successful when, contrary to the observed rarefaction curves, the EH‐adjusted rarefaction curves from the different downscaled and full datasets did not significantly differ.

## 3 RESULTS

### 3.1 From observed to EH‐adjusted rarefaction curves

**FIGURE 2:**
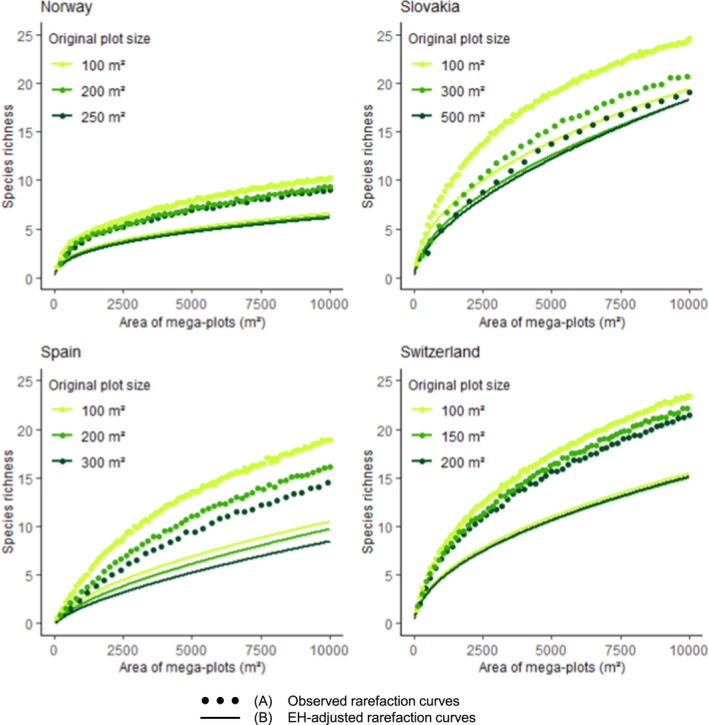
Observed (a) and EH‐adjusted (b) sample‐based rarefaction curves of each country. Different colors represent different datasets (downscaled and full plot size). However, colors are not representative of the same plot sizes across countries as their original plot size differs. Each point in (a) represents the mean SR of the 500 mega‐plots of a given size for a given dataset. EH‐adjusted rarefaction curves in (b) represent model predictions along the area gradient with no EH

**FIGURE 3:**
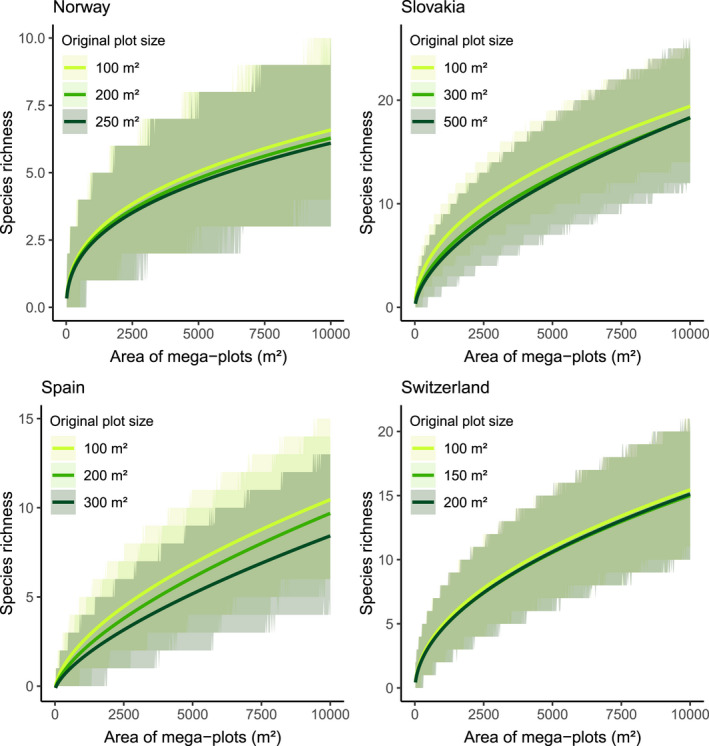
Validation: for each country, EH‐adjusted rarefaction curves, along with the corresponding 95% bootstrap prediction intervals. This figure represents a zoomed‐in version of the EH‐adjusted rarefaction curves represented in Figure 2b. The SR axis varies between countries. Different colors represent different datasets (downscaled and full plot size). Colors are not representative of the same plot sizes across countries as their original plot sizes differ. Note that the step‐like appearance of the prediction intervals relates to the fact that SR by definition is an integer as it represents count data

In all countries, we observed steeper sample‐based rarefaction curves (SR increased faster along the area gradient) when plot size was smaller (dotted lines Figure 2). Estimates of the CMP models are presented in Table S1 in the Appendix. In all countries, the EH‐adjusted rarefaction curves were less steep than the observed rarefaction curves (solid lines Figure 2). The difference between the curves of the downscaled and full datasets was markedly reduced in the EH‐adjusted rarefaction curves compared to the original ones. In all countries, the EH‐adjusted rarefaction curves from the various downscaled and full datasets did not significantly differ (Figure 3). However, the EH‐adjusted rarefaction curves of the different datasets of Switzerland and Norway were closer than the ones of Spain and Slovakia.

### 3.2 Comparison of SR between countries

**FIGURE 4:**
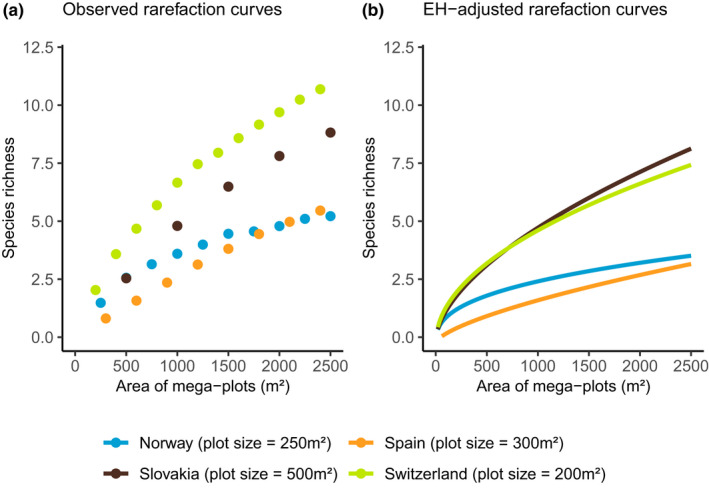
(a) Observed and (b) EH‐adjusted rarefaction curves of Norway, Slovakia, Spain, and Switzerland. The spacing between the points of each curve differs from one country to another as their plot sizes differ. EH‐adjusted curves represent predictions from the models with no EH

The observed sample‐based rarefaction curve of Switzerland was the steepest of all countries, followed by Slovakia. That would indicate, without any consideration for differences in plot sizes, that Switzerland has the highest SR of all four countries for any given area (Figure 4a). However, the EH‐adjusted rarefaction curves of Switzerland and Slovakia were closely matching (Figure 4b). The observed rarefaction curve of Norway was steeper than that of Spain for small areas, but leveled off earlier so that both countries reached a similar SR around 2000 m² (Figure 4a). This pattern persisted in the EH‐adjusted rarefaction curves (Figure 4b). At 500 m², observed rarefaction curves indicated similar SR in Slovakia and Norway, while the EH‐adjusted rarefaction curves showed a higher SR in Slovakia than in Norway.

## 4 DISCUSSION

Getting a comprehensive picture of biodiversity – more particularly SR – across different ecosystems, regions, or countries often implies combining multiple inventory datasets. However, as SR is strongly related to the area that is sampled, comparisons between inventories are hampered by differences in plot size. This aspect is not always acknowledged, which can result in misleading patterns of SR persisting in politically relevant reports such as “The State of Europe’s Forests” (FOREST EUROPE, 2020). Indeed, this report compares the SR of forests in European countries without controling for how these measures of SR are obtained in each of these countries. Our study takes the example of NFIs from Norway, Slovakia, Spain, and Switzerland to present and test a methodological framework accounting for the plot size‐dependent EH introduced when building rarefaction curves, thus enabling direct SR comparisons between inventories even when plot sizes differ.

### 4.1 Accounting for EH to enable SR comparisons between inventories using different plot sizes

As expected, we found that EH‐adjusted rarefaction curves were less steep than their corresponding observed rarefaction curves. The EH‐adjusted curves represent the relationship between SR and area assuming the sampled area is essentially environmentally homogeneous. The validation process showed that our method was successful in all countries, but performed best in Norway and Switzerland. This could indicate that the EH of Spain and Slovakia was not as well captured by the environmental variables we used as in the other countries. Additionally, species composition and diversity in Slovakia strongly differ between regions, mainly as a result of management legacies. For instance, the natural beech–fir forests in the Orava and Kysuce regions have been widely replaced by spruce monocultures. Species composition is therefore more homogeneous than in other regions, regardless of the environmental conditions these forests are subject to. In the mountainous Slovakian regions, however, species composition and environmental conditions are very diverse due to a highly complex orography. The relationship between SR and EH thus likely varies between regions, and the global databases used to extract environmental variables might not be able to capture this small‐scale variability. This could explain why our method did not perform as well in Slovakia as in the other countries.

For any specific area, forests in Spain had a rather low SR compared to the other countries. This could be because most of the forest ecosystems are located in the Mediterranean biogeographical region that dominates the Iberian Peninsula, characterized by a low crown cover, high shrub, and herbaceous plant diversity, and where only few tree species are adapted to the harsh conditions prevalent in some forest types (such as junipers forests, palm stands, carob tree forests, or strawberry tree forests). Dehesas (agrosilvopastoral systems), relatively frequent in the Mediterranean area, are also associated with reduced SR (Martín‐Queller et al., 2011). Furthermore, part of the country was reforested with monocultures over the last decades, artificially decreasing the SR of these planted forests. In Mediterranean areas, there are also numerous companion tree species that cannot reach large diameters. This causes an underestimation of SR when using small area plots or concentric plot designs that apply a caliper threshold. Therefore, the Spanish NFI also measures total tree richness in 25‐m‐radius plots that in addition to sample trees also includes regeneration and trees below the caliper threshold. The resulting total SR is on average four species, indicating that reliable SR estimations in Mediterranean forests should not solely rely on SR of sample trees.

Slovakia and Switzerland are both on average subject to less extreme climates than Norway and Spain that are located at the extremes of the European climate range. Less extreme conditions are often better suited to the local cohabitation of more species, which could explain why the EH‐adjusted rarefaction curves of Slovakia and Switzerland indicated a higher SR for any given area. Additionally, Switzerland and Slovakia might be more environmentally heterogeneous than Spain and Norway for similar areas, which could further explain the faster accumulation of SR over area. Contrary to Norway where EH‐adjusted rarefaction curves leveled off rapidly, SR kept increasing further along the area gradient in Spain, suggesting that higher SR values could potentially be attained if area was further increased. This could result from the lower total number of species (gamma diversity) in Norway than in Spain (29 and 126, respectively), most likely due to the larger area that has been sampled (number of plots multiplied by plot size) in Spain than in Norway. The few Norwegian species are more equally spread over the entire country and consequently, the total species pool is quickly reached. In Spain, the variety of ecosystems is much larger, although the number of species able to reach the DBH threshold is low. Consequently, many different ecosystems must be sampled (and aggregated) to reach the entire species pool, making both the observed and EH‐adjusted curves less steep and more gradual than in Norway.

Comparing rarefaction curves of countries before or after adjusting for differences in plot size led to different conclusions. For instance, the observed curve of Switzerland was steeper than that of Slovakia, which uses larger plots. However, once the plot size difference was accounted for by removing the EH between aggregated plots, the EH‐adjusted rarefaction curves of Switzerland and Slovakia were similar, suggesting that both countries had, for any specific area, similar levels of SR. However, this could also be affected by the difference in the performance of the method in Switzerland and Slovakia. Similarly, without accounting for plot size differences, the Slovakian observed rarefaction curve had SR values higher but relatively close to those of both Spain and Norway. After removing the effect of EH, this difference was much larger. These results showed that accounting for plot size differences is crucial when comparing SR between inventories. Our methodological framework could enable more reliable comparisons between SR of inventories using different plot sizes. For instance, the State of Europe’s Forests (FOREST EUROPE, 2020) that reports on the share of European countries presenting a given SR does so without consideration of plot size differences. Instead, our method would allow, for example, the reporting of comparable estimates of mean SR observed per hectare. Furthermore, our method is not bound to NFIs or to the diversity of tree species, but is applicable to other count data such as functional or phylogenetic diversity, thus adding value to already existing datasets.

### 4.2 Limitations

Our method relies on several assumptions. First, we built rarefaction curves such that each aggregation step was independent of the previous ones. Plots were randomly selected from the entire plot pool, thus not building on previously aggregated plots. Traditionally, rarefaction curves are based on trajectories, where at each step a plot is randomly selected and added to the already aggregated ones from the previous step (Gotelli & Colwell, 2001). The intention for building such trajectories has often been to approximate the species–area relationship in one homogeneous ecosystem or area such as an agricultural field or a lake. Since plots in such well‐defined and spatially constrained ecosystems are not independent, a trajectory‐wise aggregation is appropriate. In our case, where even the closest NFI plots are located in different forest stands, we deemed the random aggregation more appropriate to reflect the independence of the aggregated plots.

Second, our approach relies on the assumption that EH is a key factor driving differences in species composition between plots (also termed species turnover or beta diversity). However, different environmental conditions between plots might not always translate into different species compositions. This needs to be considered when interpreting the country‐specific degree to which our method adjusts rarefaction curves, and may explain why the proposed method works better in some countries than in others, for instance, as a result of forest management. Furthermore, our method assumes that the environmental variables used are indeed related to differences in SR between aggregated plots. The quality and resolution of the environmental data might not be sufficient to describe ecologically meaningful differences across plots. Additionally, other variables such as maximum temperature or summer precipitation could be better suited for certain regions or species groups. This aspect should be further investigated to better understand which variables best capture EH in each country, and how much each EH measure contributes to SR.

Third, in regions where the tree species composition has strongly been modified by forest management, e.g., by creating monospecific plantations, environmental variables might be meaningless in explaining differences in SR between aggregated plots. In other words, our method is expected to perform best in forests with native species mixes. Unfortunately, the forest naturalness level could hardly be accounted for by using variables related to forest management. Indeed, management practices often target certain species and can both increase and decrease SR depending on the treatments applied.

### 4.3 Additional confounding factors resulting from sampling design differences

Sampling design characteristics other than plot size might affect how many species are recorded in a plot. In NFIs, the DBH threshold from which trees are measured varies across countries. We artificially harmonized this threshold to match the highest one (12 cm). This might have affected SR differently in each country, as growing conditions can greatly vary between regions and along environmental gradients. Consequently, our rather high DBH threshold may hardly be reached even by mature trees where environmental conditions are limiting tree growth. This certainly occurs in Spain, and possibly in Northern Norway or in the Swiss and Slovak mountainous areas where the climate is cold and growing seasons are short. Additionally, slowly growing species – even when able to grow beyond that threshold – are less likely to be detected. Instead of using the same DBH threshold across inventories, our approach could be tested with country‐ or region‐specific DBH thresholds adapted to the prevalent growing conditions and species composition. Furthermore, our approach is applicable to inventories based on both fixed‐area and concentric circles plots, but not to inventories using angle count sampling such as in Germany, as this design is not area‐based.

### 4.4 Broader implications

By allowing reliable SR comparisons between inventories using different plot sizes, our method could be used to compile data from several monitoring programs to report on large‐scale biodiversity patterns. Such comparisons are relevant both within countries when several inventories using different plot sizes are in place, e.g., for different regions or ecosystems, as well as internationally, where differences in plot sizes are the rule rather than the exception. Our approach could enable data‐driven, large‐scale comparisons of SR, for example, across Europe. Such attempts would involve the following steps: (1) build rarefaction curves by aggregating plots for each inventory of interest, (2) perform inventory‐specific CMP regressions to quantify EH effects, (3) predict SR with no EH along the area gradient, and (4) use these predictions to translate to a common plot size. Furthermore, our method could be used to provide private owners or land managers with a tool informing them on the number of species they can expect to find in their land, given its area and location. This tool could help planning and making decisions on the potential necessity to take actions aiming at increasing biodiversity.

Our approach focuses on SR, but could be applicable to any other plot‐based count data having a non‐linear relationship to area, such as the number of veteran trees, structural elements, or microhabitats. Furthermore, our method is not bound to NFIs, but could be applied to any other plot‐based inventory or monitoring program. Given the large amount of biodiversity datasets that have been put together over the last decades, our method is in line with the growing interest of the scientific community in big data.

Further steps building on our approach could involve investigating beta diversity, or other related biodiversity indicators such as functional or phylogenetical diversity. Our models based on the quantification of EH and its effect on rarefaction curves could also be further developed to investigate the effect of other environmental factors, explore further to which level these EH variables are affecting SR depending on the region or country, and compare their effects. Consequently, large compiled and plot‐size corrected datasets could enable unseen research, e.g., on the environmental determinants of SR across large areas, maybe even entire continents, including their potential trends over time. Our approach has therefore a high applicability and transferability that could be further pursued.

## AUTHOR CONTRIBUTION

**Jeanne Portier:** Conceptualization (lead); Formal analysis (lead); Investigation (lead); Methodology (lead); Validation (lead); Visualization (lead); Writing – original draft (lead); Writing – review & editing (lead). **Florian Zellweger:** Conceptualization (equal); Data curation (lead); Investigation (equal); Writing – original draft (supporting); Writing – review & editing (supporting). **Jürgen Zell:** Formal analysis (equal); Investigation (equal); Methodology (equal); Validation (equal); Writing – original draft (equal); Writing – review & editing (equal). **Iciar Alberdi Asensio** Data curation (equal); Resources (equal); Writing – original draft (supporting); Writing – review & editing (supporting). **Michal Bošeľa:** Data curation (equal); Resources (equal); Writing – original draft (supporting); Writing – review & editing (supporting). **Johannes Breidenbach:** Data curation (equal); Resources (equal); Writing – original draft (supporting); Writing – review & editing (supporting). **Vladimir Seben:** Data curation (equal); Resources (equal); Writing – original draft (supporting); Writing – review & editing (supporting). **Raphael Wuest:** Conceptualization (equal); Formal analysis (equal); Funding acquisition (lead); Investigation (equal); Methodology (equal); Project administration (lead); Supervision (lead); Validation (equal); Writing – original draft (equal); Writing – review & editing (equal). **Brigitte Rohner:** Conceptualization (equal); Formal analysis (equal); Funding acquisition (lead); Investigation (equal); Methodology (equal); Project administration (lead); Supervision (lead); Validation (equal); Writing – original draft (equal); Writing – review & editing (equal).

## CONFLICT OF INTEREST

The authors declare no conflict of interest.

## Supporting information

Supplementary Material

## Data Availability

The aggregated full and downscaled datasets at the mega‐plot level, including EH variables, are stored in the EnviDat repository (https://doi.org/10.16904/envidat.318) and are publicly available. Raw NFI data are not publicly available as the conditions regarding their availability vary from one country to another and their accessibility is restricted by law. Contact details for raw NFI data requests can be found in the ENFIN website (http://enfin.info/).

## References

[ece38965-bib-0001] Alberdi, I. , Cañellas, I. , & Vallejo Bombín, R. (2017). The Spanish National Forest Inventory: History, development, challenges and perspectives. Pesquisa Florestal Brasileira, 37, 361. 10.4336/2017.pfb.37.91.1337

[ece38965-bib-0002] Ballabio, C. , Lugato, E. , Fernández‐Ugalde, O. , Orgiazzi, A. , Jones, A. , Borrelli, P. , Montanarella, L. , & Panagos, P. (2019). Mapping LUCAS topsoil chemical properties at European scale using Gaussian process regression. Geoderma, 355, 113912. 10.1016/j.geoderma.2019.113912 31798185 PMC6743211

[ece38965-bib-0003] Beers, T. W. , Dress, P. E. , & Wensel, L. C. (1966). Notes and observations: Aspect transformation in site productivity research. Journal of Forestry, 64, 691–692.

[ece38965-bib-0004] Breidenbach, J. , Granhus, A. , Hylen, G. , Eriksen, R. , & Astrup, R. (2020). A century of National Forest Inventory in Norway – informing past, present, and future decisions. *Forest* . Ecosystems, 7, 7–46. 10.1186/s40663-020-00261-0 PMC7366156 32834905

[ece38965-bib-0025] Chirici, G. , Winter, S. , & McRoberts, R. E. (Eds.). (2011). National forest inventories: Contributions to forest biodiversity assessments (Vol. 20). Springer Science & Business Media.

[ece38965-bib-0005] Corona, P. , Chirici, G. , McRoberts, R. E. , Winter, S. , & Barbati, A. (2011). Contribution of large‐scale forest inventories to biodiversity assessment and monitoring. Forest Ecology and Management, 262, 2061–2069. 10.1016/j.foreco.2011.08.044

[ece38965-bib-0006] Crist, T. O. , & Veech, J. A. (2006). Additive partitioning of rarefaction curves and species‐area relationships: Unifying α‐, β‐ and γ‐diversity with sample size and habitat area. Ecology Letters, 9, 923–932. 10.1111/j.1461-0248.2006.00941.x 16913935

[ece38965-bib-0007] Dengler, J. (2009). Which function describes the species‐area relationship best? A review and empirical evaluation. Journal of Biogeography, 36, 728–744. 10.1111/j.1365-2699.2008.02038.x

[ece38965-bib-0008] Dengler, J. , Matthews, T. J. , Steinbauer, M. J. , Wolfrum, S. , Boch, S. , Chiarucci, A. , Conradi, T. , Dembicz, I. , Marcenò, C. , García‐Mijangos, I. , Nowak, A. , Storch, D. , Ulrich, W. , Campos, J. A. , Cancellieri, L. , Carboni, M. , Ciaschetti, G. , De Frenne, P. , Dolezal, J. , … Biurrun, I. (2020). Species–area relationships in continuous vegetation: Evidence from Palaearctic grasslands. Journal of Biogeography, 47, 72–86. 10.1111/jbi.13697

[ece38965-bib-0009] Drakare, S. , Lennon, J. J. , & Hillebrand, H. (2006). The imprint of the geographical, evolutionary and ecological context on species‐area relationships. Ecology Letters, 9, 215–227. 10.1111/j.1461-0248.2005.00848.x 16958886

[ece38965-bib-0010] EU‐DEM . (2018). EU‐Digital Elevation Model (DEM).

[ece38965-bib-0011] European Commission . (2021). Communication from the commission to the European parliament, the council, the European Economic and Social Committee and the committee of the regions – New EU forest strategy for 2030.

[ece38965-bib-0012] European Environment Agency . (2016). Biogeographical regions.

[ece38965-bib-0013] Field, R. , Hawkins, B. A. , Cornell, H. V. , Currie, D. J. , Diniz‐Filho, J. A. F. , Guégan, J. F. , Kaufman, D. M. , Kerr, J. T. , Mittelbach, G. G. , Oberdorff, T. , O’Brien, E. M. , & Turner, J. R. G. (2009). Spatial species‐richness gradients across scales: A meta‐analysis. Journal of Biogeography, 36, 132–147. 10.1111/j.1365-2699.2008.01963.x

[ece38965-bib-0014] FOREST EUROPE (2020). State of Europe’s Forests 2020. 394.

[ece38965-bib-0015] Gotelli, N. J. , & Colwell, R. K. (2001). Quantifying biodiversity: Procedures and pitfalls in the measurement and comparison of species richness. Ecology Letters, 4, 379–391. 10.1046/j.1461-0248.2001.00230.x

[ece38965-bib-0016] Gotelli, N. J. , & Colwell, R. K. (2011). Chapter 4: Estimating species richness. In A. E. Magurran & B. J. McGill (Eds.), Biological diversity: Frontiers in measurement and assessment (pp. 39–54). Oxford University Press.

[ece38965-bib-0017] Güler, B. , Jentsch, A. , Apostolova, I. , Bartha, S. , Bloor, J. M. G. , Campetella, G. , Canullo, R. , Házi, J. , Kreyling, J. , Pottier, J. , Szabó, G. , Terziyska, T. , Uğurlu, E. , Wellstein, C. , Zimmermann, Z. , & Dengler, J. (2016). How plot shape and spatial arrangement affect plant species richness counts: implications for sampling design and rarefaction analyses. Journal of Vegetation Science, 27, 692–703. 10.1111/jvs.12411

[ece38965-bib-0018] Guyot, V. , Castagneyrol, B. , Vialatte, A. , Deconchat, M. , & Jactel, H. (2016). Tree diversity reduces pest damage in mature forests across Europe. Biology Letters, 12. 10.1098/rsbl.2015.1037 PMC4881340 27122011

[ece38965-bib-0019] Hengl, T. , Mendes de Jesus, J. , Heuvelink, G. B. M. , Ruiperez Gonzalez, M. , Kilibarda, M. , Blagotić, A. , Shangguan, W. , Wright, M. N. , Geng, X. , Bauer‐Marschallinger, B. , Guevara, M. A. , Vargas, R. , MacMillan, R. A. , Batjes, N. H. , Leenaars, J. G. B. , Ribeiro, E. , Wheeler, I. , Mantel, S. , & Kempen, B. (2017). SoilGrids250m: Global gridded soil information based on machine learning. PLoS One, 12, e0169748. 10.1371/journal.pone.0169748 28207752 PMC5313206

[ece38965-bib-0020] Hill, J. L. , Curran, P. J. , & Foody, G. M. (1994). The effect of sampling on the species‐area curve. Global Ecology and Biogeography Letters, 4, 97. 10.2307/2997435

[ece38965-bib-0021] IPBES . (2019). In S. Díaz , J. Settele , E. S. Brondízio , H. T. Ngo , M. Guèze , J. Agard , A. Arneth , P. Balvanera , K. A. Brauman , S. H. M. Butchart , K. M. A. Chan , L. A. Garibaldi , K. Ichii , J. Liu , S. M. S. Díaz , J. Settele , E. S. Brondízio , H. T. Ngo , & M. Guèze (Eds.), Summary for policymakers of the global assessment report on biodiversity and ecosystem services of the Intergovernmental Science‐Policy Platform on Biodiversity and Ecosystem Services. IPBES secretariat.

[ece38965-bib-0022] Karger, D. N. , Conrad, O. , Böhner, J. , Kawohl, T. , Kreft, H. , Soria‐Auza, R. W. , Zimmermann, N. E. , Linder, H. P. , & Kessler, M. (2017). Climatologies at high resolution for the earth’s land surface areas. Scientific Data, 4, 170122. 10.1038/sdata.2017.122 28872642 PMC5584396

[ece38965-bib-0023] Lengyel, S. , Kobler, A. , Kutnar, L. , Framstad, E. , Henry, P. Y. , Babij, V. , Gruber, B. , Schmeller, D. , & Henle, K. (2008). A review and a framework for the integration of biodiversity monitoring at the habitat level. Biodiversity and Conservation, 17, 3341–3356. 10.1007/s10531-008-9359-7

[ece38965-bib-0024] Martín‐Queller, E. , Gil‐Tena, A. , & Saura, S. (2011). Species richness of woody plants in the landscapes of Central Spain: The role of management disturbances, environment and non‐stationarity. Journal of Vegetation Science, 22, 238–250. 10.1111/j.1654-1103.2010.01242.x

[ece38965-bib-0026] Oliver, T. H. , Heard, M. S. , Isaac, N. J. B. , Roy, D. B. , Procter, D. , Eigenbrod, F. , Freckleton, R. , Hector, A. , Orme, C. D. L. , Petchey, O. L. , Proença, V. , Raffaelli, D. , Suttle, K. B. , Mace, G. M. , Martín‐López, B. , Woodcock, B. A. , & Bullock, J. M. (2015). Biodiversity and resilience of ecosystem functions. Trends in Ecology and Evolution, 30, 673–684. 10.1016/j.tree.2015.08.009 26437633

[ece38965-bib-0027] R Core Team . (2020). R: A language and environment for statistical computing. R Foundation for Statistical Computing. https://www.R‐project.org/

[ece38965-bib-0028] Sellers, K. F. , & Raim, A. (2016). A flexible zero‐inflated model to address data dispersion. Computational Statistics & Data Analysis, 99, 68–80. 10.1016/j.csda.2016.01.007

[ece38965-bib-0029] Sellers, K. F. , & Shmueli, G. (2010). A flexible regression model for count data. Annals of Applied Statistics, 4, 943–961. 10.1214/09-AOAS306

[ece38965-bib-0030] Shmueli, G. , Minka, T. P. , Kadane, J. B. , Borle, S. , & Boatwright, P. (2005). A useful distribution for fitting discrete data: Revival of the Conway‐Maxwell‐Poisson distribution. Journal of the Royal Statistical Society Series C: Applied Statistics, 54, 127–142.

[ece38965-bib-0031] Silva Pedro, M. , Rammer, W. , & Seidl, R. (2015). Tree species diversity mitigates disturbance impacts on the forest carbon cycle. Oecologia, 177, 619–630. 10.1007/s00442-014-3150-0 25526843

[ece38965-bib-0032] Stein, A. , Gerstner, K. , & Kreft, H. (2014). Environmental heterogeneity as a universal driver of species richness across taxa, biomes and spatial scales. Ecology Letters, 17, 866–880. 10.1111/ele.12277 24751205

[ece38965-bib-0033] Steinmann, K. , Eggenberg, S. , Wohlgemuth, T. , Linder, H. P. , & Zimmermann, N. E. (2011). Niches and noise‐Disentangling habitat diversity and area effect on species diversity. Ecological Complexity, 8, 313–319. 10.1016/j.ecocom.2011.06.004

[ece38965-bib-0034] Tittensor, D. P. , Micheli, F. , Nyström, M. , & Worm, B. (2007). Human impacts on the species–area relationship in reef fish assemblages. Ecology Letters, 10, 760–772. 10.1111/j.1461-0248.2007.01076.x 17663709

[ece38965-bib-0035] Tomppo, E. , T. Gschwantner , M. Lawrence , & R. E. McRoberts (Eds.) (2010). National forest inventories. Pathways for common reporting. Dordrecht.

[ece38965-bib-0036] Vidal, C. , I. A. Alberdi , L. Hernández Mateo , & J. J. Redmond (Eds.). (2016). National forest inventories. Assessment of wood availability and use. Springer International Publishing.

[ece38965-bib-0037] Vidal, C. , Alberdi, I. , Redmond, J. , Vestman, M. , Lanz, A. , & Schadauer, K. (2016). The role of European National Forest Inventories for international forestry reporting. Annals of Forest Science, 73, 793–806. 10.1007/s13595-016-0545-6

[ece38965-bib-0038] Winter, S. , Chirici, G. , McRoberts, R. E. , Hauk, E. , & Tomppo, E. (2008). Possibilities for harmonizing national forest inventory data for use in forest biodiversity assessments. Forestry, 81, 33–44. 10.1093/forestry/cpm042

