# Supplementary material for: Plot size matters: Toward comparable species richness estimates across plot‐based inventories
Source: Ecol Evol. 2022 Jun 12;12(6):e8965. doi: 10.1002/ece3.8965 (PMC9189332; doi:10.1002/ece3.8965)
Supplement: Supplementary file 1 — Supplementary Material [file ECE3-12-e8965-s001.docx]

# Appendix

Table S1: Estimates and standard error (SE) of parameters from the CMP models of each downscaled and full datasets of each country.

| Country | | | Norway | | | | Slovakia | | | | |
| --- | --- | --- | --- | --- | --- | --- | --- | --- | --- | --- | --- |
| Plot size (m²) | | | **100** | **150** | **200** | **250** | **100** | **200** | **300** | **400** | **500** |
| λ | $\beta_{0}$ | Estimate | -2.881 | -2.940 | -2.954 | -2.998 | -2.555 | -3.172 | -3.377 | -3.391 | -3.479 |
|  |  | SE | 0.036 | 0.045 | 0.052 | 0.059 | 0.026 | 0.041 | 0.052 | 0.059 | 0.067 |
|  | Log(A) ($\beta_{1})$ | Estimate | 1.042 | 1.000 | 0.939 | 0.918 | 1.144 | 1.080 | 0.980 | 0.895 | 0.860 |
|  |  | SE | 0.008 | 0.009 | 0.010 | 0.011 | 0.008 | 0.010 | 0.012 | 0.012 | 0.013 |
|  | H_clim_ $(\beta_{2})$ | Estimate | 0.062 | 0.072 | 0.068 | 0.067 | 0.002 | -0.001 | -0.002 | -0.012 | -0.023 |
|  |  | SE | 0.004 | 0.005 | 0.006 | 0.006 | 0.003 | 0.004 | 0.004 | 0.005 | 0.005 |
|  | H_topo_ $(\beta_{3})$ | Estimate | 0.042 | 0.045 | 0.048 | 0.065 | 0.048 | 0.042 | 0.045 | 0.044 | 0.044 |
|  |  | SE | 0.004 | 0.005 | 0.006 | 0.006 | 0.003 | 0.004 | 0.005 | 0.005 | 0.006 |
|  | H_soil_ $(\beta_{4})$ | Estimate | 0.012 | 0.010 | 0.005 | 0.006 | 0.062 | 0.067 | 0.057 | 0.059 | 0.056 |
|  |  | SE | 0.004 | 0.005 | 0.005 | 0.006 | 0.003 | 0.004 | 0.004 | 0.004 | 0.005 |
|  | H_BA_ $(\beta_{5})$ | Estimate | 0.119 | 0.124 | 0.125 | 0.122 | -0.026 | -0.034 | -0.046 | -0.044 | -0.049 |
|  |  | SE | 0.004 | 0.004 | 0.005 | 0.005 | 0.003 | 0.004 | 0.004 | 0.004 | 0.005 |
|  | H_res_ $(\beta_{6})$ | Estimate | -0.056 | -0.065 | -0.060 | -0.066 | 0.050 | 0.052 | 0.046 | 0.052 | 0.011 |
|  |  | SE | 0.003 | 0.004 | 0.005 | 0.005 | 0.002 | 0.003 | 0.004 | 0.004 | 0.004 |
| ν | | Estimate | 2.849 | 2.729 | 2.528 | 2.454 | 2.452 | 2.156 | 1.831 | 1.603 | 1.487 |
|  |  | SE | 0.018 | 0.021 | 0.023 | 0.025 | 0.016 | 0.019 | 0.020 | 0.020 | 0.021 |

| Country | | | Spain | | | | | Switzerland | | |
| --- | --- | --- | --- | --- | --- | --- | --- | --- | --- | --- |
| Plot size (m²) | | | **100** | **150** | **200** | **250** | **300** | **100** | **150** | **200** |
| λ | $\beta_{0}$ | Estimate | -5.486 | -5.998 | -6.360 | -6.629 | -6.591 | -3.01 | -3.16 | -3.11 |
|  |  | SE | 0.042 | 0.055 | 0.067 | 0.078 | 0.085 | 0.028 | 0.036 | 0.04 |
|  | Log(A) ($\beta_{1})$ | Estimate | 1.323 | 1.325 | 1.309 | 1.308 | 1.260 | 1.16 | 1.09 | 1.02 |
|  |  | SE | 0.009 | 0.011 | 0.012 | 0.014 | 0.015 | 0.008 | 0.009 | 0.01 |
|  | H_clim_ $(\beta_{2})$ | Estimate | 0.059 | 0.065 | 0.072 | 0.070 | 0.079 | 0.04 | 0.04 | 0.03 |
|  |  | SE | 0.003 | 0.004 | 0.005 | 0.005 | 0.006 | 0.003 | 0.004 | 0.004 |
|  | H_topo_ $(\beta_{3})$ | Estimate | 0.052 | 0.055 | 0.053 | 0.042 | 0.058 | 0.06 | 0.07 | 0.07 |
|  |  | SE | 0.003 | 0.004 | 0.004 | 0.005 | 0.005 | 0.003 | 0.004 | 0.004 |
|  | H_soil_ $(\beta_{4})$ | Estimate | 0.022 | 0.022 | 0.019 | 0.003 | 0.025 | 0.08 | 0.08 | 0.08 |
|  |  | SE | 0.004 | 0.004 | 0.005 | 0.005 | 0.006 | 0.003 | 0.003 | 0.004 |
|  | H_BA_ $(\beta_{5})$ | Estimate | 0.119 | 0.123 | 0.122 | 0.118 | 1.134 | -0.05 | -0.05 | -0.05 |
|  |  | SE | 0.003 | 0.004 | 0.004 | 0.005 | 0.005 | 0.003 | 0.003 | 0.004 |
|  | H_res_ $(\beta_{6})$ | Estimate | 0.079 | 0.077 | 0.078 | -0.007 | 0.095 | 0.07 | 0.08 | 0.07 |
|  |  | SE | 0.003 | 0.004 | 0.004 | 0.004 | 0.005 | 0.003 | 0.003 | 0.003 |
| ν | | Estimate | 2.242 | 2.142 | 2.019 | 1.960 | 1.860 | 2.40 | 2.20 | 2.04 |
|  |  | SE | 0.014 | 0.017 | 0.018 | 0.020 | 0.021 | 0.02 | 0.02 | 0.02 |


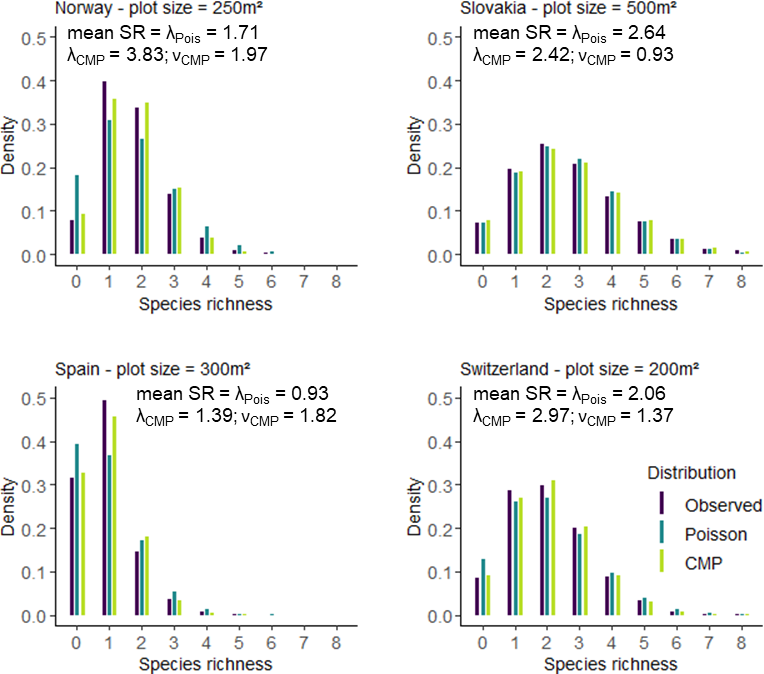


Figure A 1: Observed SR distribution in the NFI plots of each country, as well as obtained under a Poisson distribution and a CMP distribution. Values of the parameters of each distribution are also presented.
